# Are We Reaching Everyone? A Cross-Sectional Study of Telehealth Inequity in the COVID-19 Pandemic in an Urban Academic Pediatric Primary Care Clinic

**DOI:** 10.1177/00099228211045809

**Published:** 2022-01

**Authors:** Rachel B. Schenker, Meredith C. Laguna, Anobel Y. Odisho, Megumi J. Okumura, Honora Burnett

**Affiliations:** 1 University of California, San Francisco, CA, USA

**Keywords:** telehealth, telemedicine, COVID-19, SARS-CoV-2, disparities

## Abstract

The COVID-19 (coronavirus disease 2019) pandemic brought rapid expansion of pediatric telehealth to maintain patient access to care while decreasing COVID-19 community spread. We designed a retrospective, serial, cross-sectional study to investigate if telehealth implementation at an academic pediatric practice led to disparities in health care access. Significant differences were found in pre-COVID-19 versus during COVID-19 patient demographics. Patients seen during COVID-19 were more likely to be younger, White/Caucasian or Asian, English speaking, and have private insurance. They were less likely to be Black/African American or Latinx and request interpreters. Age was the only significant difference in patient demographics between in-person and telehealth visits during COVID-19. A multivariate regression showed older age as a significant positive predictor of having a video visit and public insurance as a significant negative predictor. Our study demonstrates telehealth disparities based on insurance existed at our clinic as did inequities in who was seen before versus during COVID-19.

## Introduction

Telehealth is a mechanism by which patients can access pediatricians via mobile device for either face-to-face or telephone visits.^1^ It has been used to reach patients who experience difficulties seeing their providers in person, such as those with significant disabilities or long travel times to clinic. Telehealth was not initially widely adopted due to provider and patient technological, cultural, and financial barriers. As recently as 2016, only 12% of pediatricians worked in practices utilizing telehealth.^2^ When the coronavirus disease 2019 (COVID-19) pandemic hit the United States, pediatricians were forced to make significant changes in clinical practice starting in March 2020. To maintain access to care, while allowing for social distancing and mitigating community spread of COVID-19, medical providers dramatically expanded their telehealth services.^3^ In addition, to ensure practitioners were adequately funded for this care, Medicare and Medicaid liberalized telehealth reimbursement policies. Studies estimated a 6-fold increase in telehealth appointments between March 2, 2020, and April 14, 2020.^4^

In the case of the COVID-19 pandemic, telehealth’s rapid expansion has allowed for access to medical care while minimizing exposure among patients and providers. Reports published throughout 2020 have suggested there may be unequal telehealth access across the demographic spectrum, but results have been inconsistent.^5-11^ This study furthers the conversation by investigating whether telehealth implementation in a pediatric clinic at an academic medical center led to disparities in health care access. We undertook this by comparing patient visit demographics for all visits before and after the start of the COVID-19 pandemic, and between in-person visits and telehealth visits during the COVID-19 pandemic. We then investigate factors that may be related to any disparities.

## Methods

### Setting

The University of California, San Francisco (UCSF) Pediatric Clinic at Mount Zion (Mt Zion) is a pediatric primary and acute care clinic embedded within a large, urban academic medical center. Historically, it been subdivided into well care (for well-child checks and health care maintenance) and acute care (for urgent complaints). Prior to March 2020, nearly all visits were conducted in person. The clinic is staffed by medical assistants, nurses, social workers, 42 UCSF pediatric residents, and 20 UCSF faculty primary care pediatricians. It is the medical home for approximately 13 000 children (specific demographics listed under results). All visits are documented in the Epic electronic medical record (EMR).

### Clinic Changes During the COVID-19 Pandemic

**Figure 1.**
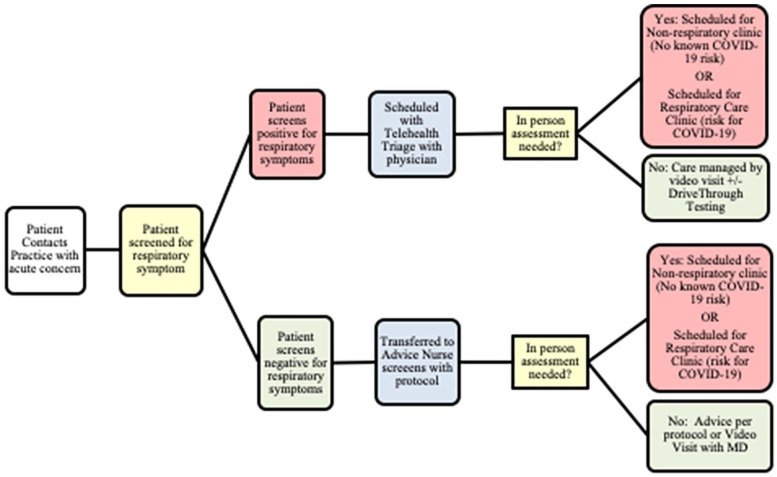
Workflow for nurses scheduling visits during COVID-19.

**Figure 2.**
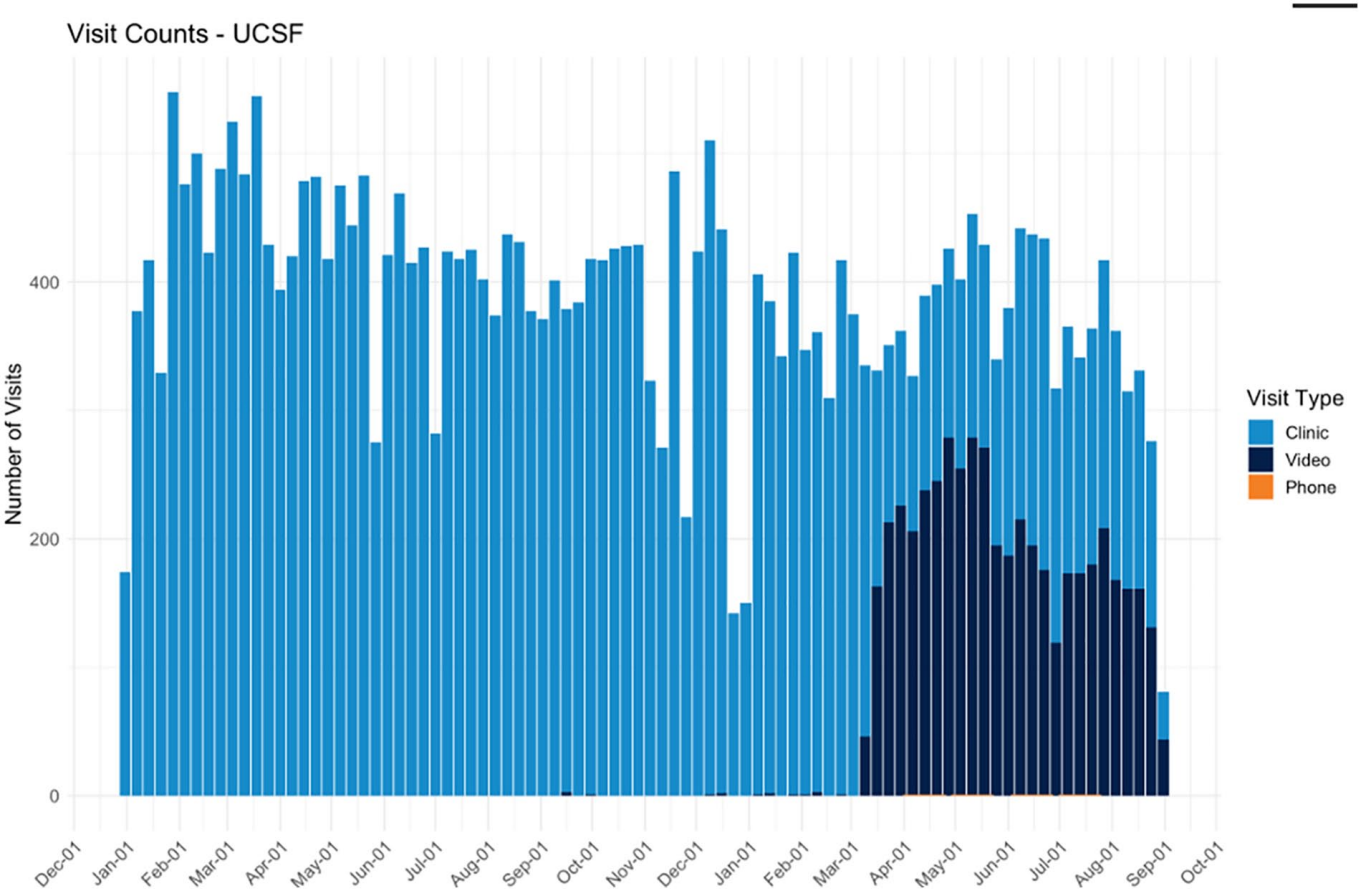
All visits by type from January 2019 to September 2020. Appointment Dates (2019-2020).

In March 2020, as concern for community spread of COVID-19 grew, Mt Zion began strengthening its telehealth presence. San Francisco mandated “Shelter in Place” (SIP) on March 16, 2020, which forced businesses to close and essential services such as health care to enact strict guidelines on in-person visits. On the same day, Mt Zion acute care was sub-divided into 3 new clinics to help identify and isolate patients with possible COVID-19: (1) non-respiratory acute care (in-person and telehealth visits for acute complaints unlikely related to COVID-19), (2) respiratory teletriage (telehealth visits for acute symptoms such as fever/cough, that could be due to COVID-19), and (3) respiratory clinic (in-person visits for patients seen in teletriage who were then deemed to need in person care). Patients with acute complaints called the nurse advice line for symptom screening (Figure 1). Based on the results of the screen, a nurse would then either provide advice or determine that the patient required physician attention and assign him/her to 1 of the 3 clinics. Well visits were still offered in person for visits typically requiring vaccines other than flu, and via telehealth for non-vaccine visits with in-person follow-up as needed. Rollout was swift: Mt Zion transitioned from pre-COVID-19 having 2 providers conducting less than 10% of their visits via telehealth, to late March 2020, having all 20 attending physicians conducting greater than 50% of well and acute care visits via telehealth (Figure 2). By April 2020, 42 residents with continuity clinic at Mt Zion were also utilizing telehealth for primary care.

### Study Design and Analysis

We designed a retrospective, pre-post cross-sectional cohort study to investigate patterns in Mt Zion clinic visit volume and demographics of patients served. To this end, we collected data on all Mt Zion appointments for 2 time periods: (1) pre-COVID-19 (March 15, 2019, to August 31, 2019) and (2) during COVID-19 (March 15, 2020, to August 31, 2020). To capture the full ramp-up of telehealth, we collected 5 months’ worth of data, starting the day of San Francisco’s SIP directive. Initially, the SIP orders required individuals to “stay in their residences except for essential needs like grocery shopping, working in essential businesses, providing essential government functions, or engaging in essential travel.”^12^ Our pre-COVID comparison was the same 5-month calendar period in 2019 to account for seasonality of pediatric illness.

Completed Mt Zion well-child and acute appointments were included during the selected date ranges. All canceled and no-show appointments were excluded. Telephone visits were also excluded as they were not captured in the EMR as it was not possible to distinguish between regular phone calls or cancelled/failed video visits. Patients were identified as video visit capable if they had a video visit during the post-COVID study period. All multivariate and patient demographic data were at the patient level unless otherwise indicated as visit level. For Table 1, we indicated well child visit versus acute care based on the visit type at first entry into the cohort period.

**Table 1.** Patient Demographics Pre-COVID-19 and During COVID-19.

	Overall visits			Well visits^a^			Acute visits^a^		
	March-August 2019	March-August 2020	*P*	March-August 2019	March-August 2020	*P*	March-August 2019	March-August 2020	*P*
Unique patients seen	6576	5385		4661	3617		2582	2678	
Age (median, IQR)	4.3 (1.2-10.4)	3 (0.8-8.5)	<.001	3.9 (1-9.4)	1.6 (0.5-5.7)	<.001	3.3 (0.7-9.3)	3.1 (0.7-9.4)	
Sex (% male)	51.1%	52.5%	.577	51.2%	53.3%	.598	52.6%	51.0%	.598
Race/ethnicity									
Unknown/declined	1.9%	2.8%	<.001	2.1%	2.8%	.001	1.7%	2.5%	<.001
White or Caucasian	34.5%	37%		37.1%	38.4%		34.5%	37.4%	
Black or African American	11.4%	8.7%		9.5%	7.3%		10.0%	9.2%	
Latin X	17.7%	16.5%		15.4%	13.7%		19.5%	18.7%	
Asian	21.8%	23.2%		23.1%	26.3%		22.1%	19.8%	
Native Hawaiian or Other Pacific Islander	0%	0%		0%	0%		0%	0%	
American Indian or Alaskan Native	0.2%	0.2%		0.1%	0.2%		0.2%	0.2%	
Multiethnicity	3.8%	3.9%		4.0%	3.9%		4.6%	4.5%	
Other	7.8%	6.9%		7.8%	6.9%		6.7%	7.0%	
English primary language	95.5%	96.8%	<.001	95.9%	97.5%	<.001	94.9%	96.3%	<.001
Interpreter requested	4.8%	3.5%	<.001	4.4%	2.7%	<.001	5.5%	3.7%	<.001
Insurance type									
Commercial (%)	66.8%	74.6%	<.001	71.8%	80.4%	<.001	67.5%	72.6%	.102
Public (%)	30.8%	23.3%		26.1%	18%		30.8%	25.4%	
Self-pay (%)	1.9%	1.6%		1.7%	1.2%		1.4%	1.4%	

a Well visit and acute care visit designation was based on the first visit within the cohort period. This does not reflect the total acute care nor well visit demographic.

For each appointment, we collected patient demographic data such as age, gender, race/ethnicity, primary language, request for an interpreter, insurance type, and primary visit diagnosis codes (CPT [Current Procedural Terminology]). All data were extracted from the EMR in September and October 2020.

We used χ^2^ and Wilcoxon rank sum tests to compare patient demographics before and during COVID-19. We also compared demographics of patients seen only in person versus ever seen by telehealth. We conducted a multivariate logistic regression of the 2020 visits on our primary outcome of having any video visit. Our independent variables were age, sex, insurance type, English spoken, and well child visit status. Statistical analyses were conducted in R.^13^ This study was approved by the UCSF Institutional Review Board.

## Results

A total of 6576 total unique pediatric patients (total of 9456 visits) were seen at Mt Zion during our pre-COVID-19 timeframe (between March and August 2019), and 5386 patients (total of 8674 visits) were seen during the COVID-19 timeframe (between March and August 2020).

Mt Zion’s total patient panel in 2019, pre-COVID-19, was 13 188 split between 51% male and 49% female. These patients were predominantly ages 0 to 21 years. In 2019, the clinic had a total of 22 860 primary care visits and 9028 acute care visits. Twenty-four percent of patients were publicly insured with 73% privately insured and 3% other/unknown. For race, 35% of patients were White/Caucasian, 23% Asian, 15% Latinx, 6% Black, 0.5% Native Hawaiian, 0.5% American Indian, and the rest unknown/declined. For language, 73% of patients spoke English as their primary language, 7% Spanish, 5% American Sign Language, 4% Mandarin/Cantonese (Chinese), with the rest of patients speaking a mix of Arabic, Japanese, Mongolian, Russian, and Thai. During the pandemic, 59% of patients seen had at least one video visit.

### Comparison of Patient Demographics Pre-COVID-19 and During the COVID-19 Pandemic

Significant differences were found in median age, racial self-identification, insurance type, primary spoken language, and requests for an interpreter in the population seen pre-COVID-19 versus during COVID-19 in well visits, acute visits, and overall (Table 1). Patients who completed any visit during the pandemic were younger, more likely to be White/Caucasian or Asian and less likely to be Black/African American or Latinx, more likely to be English speaking, less likely to request an interpreter, and more likely to have private insurance.

### Unadjusted Comparison of In-Person and Telehealth Patient Demographics During the COVID-19 Pandemic

The only significant difference in patient demographics between in-person and telehealth visits during the pandemic was age (Table 2). No significant differences were seen in primary spoken language, requests for an interpreter, insurance type, or racial self-identification.

**Table 2.** Differences in Patient Cohorts in Video/Clinic During COVID-19.

	Clinic	Video	*P*
Unique patients seen	2595	3727	
Age (median, interquartile range)	1 (0.2-5)	3.2 (1.2–9)	<.001
Sex (% male)	52.6%	52.3%	.143
Race/ethnicity			
Unknown/declined	3	2.4%	.078
White or Caucasian	36.8%	38.4%	
Black or African American	8.5%	7.5%	
Latinx	16.1%	16.6%	
Asian	24.5%	22.8%	
Native Hawaiian or Other Pacific Islander	0%	0%	
American Indian or Alaskan Native	0.2%	0.2%	
Multiethnicity	4.1%	4.3%	
Other	6.1%	7.2%	
English primary language	96.9%	97.1%	.531
Interpreter requested	3.4%	3%	1.000
Insurance type			
Commercial (%)	76.6%	77.2%	.218
Public (%)	21.5%	21.3%	
Self-pay (%)	1.4%	1.1%	

### Multi-Variable Regression Model Predicting Any Video Visit During the Pandemic

In a multivariate regression model, older age was a significant positive predictor of having a video visit while public insurance was a significant negative predictor. Racial identification and English-speaking status were not significant predictors (Table 3).

**Table 3.** Multivariate Model of Any Video Visit During COVID-19.

Terms	Odds ratio	95% Confidence interval
Intercept	3.175	1.888-5.397
Age	1.034	1.021-1.047
Sex		
Female	Ref	
Male	1.000	0.882-1.133
Race/ethnicity		
Unknown/declined	Ref	
White or Caucasian	1.388	0.944-2.020
Black or African American	1.002	0.649-1.537
Latinx	1.229	0.820-1.824
Asian	1.253	0.847-1.836
Native Hawaiian or Other Pacific Islander	0.959	0.422-2.255
American Indian or Alaskan Native	1.145	0.299-5.602
Multiethnicity	1.651	1.008-2.708
Other	1.534	0.928-2.383
English primary language	1.246	0.863-1.784
Insurance type		
Private	Ref	0.620-0.870
Public	0.734	
Self-pay	0.611	0.361-1.054
Other	0.388	0.175-0.884
Well visit	0.285	0.248-0.328

## Discussion

We saw a decrease of more than 1000 unique patients (~8% of our total clinic population) seen during the pandemic as compared with the same time period in the previous year. For those who did seek care, more than 40% of our pediatric patients did not have any video visit during the pandemic study period. Our results demonstrate that during the COVID-19 pandemic, patients who completed a medical visit of any kind were significantly more likely to be White or Asian and to have private insurance and speak English. When examining patients seen during the pandemic in person, versus telehealth, the only significant finding was a younger age for in-person visits. Our regression model also demonstrated older age was a positive predictor of video visits and public insurance was a negative predictor. The trend toward seeing younger patients in-person was unsurprising given our protocols prioritized in person visits for younger patients due to the need for vaccinations and more frequent growth monitoring. We found racial, insurance, and language disparities in who accessed care from Mt Zion in the first 5 months of COVID-19 pandemic and insurance disparities in the modality used to provide care (telehealth vs in-person).

We hypothesize that the differences seen in our population during the pandemic may have been due to COVID-19’s disproportionate burden on low-income families. During the pandemic, concern about contracting COVID-19, reduction in public transportation, limited child care options due to school cancellation, and difficulty getting time off work due to particularly high fear of job loss may have prevented lower-income families on public insurance from reaching out for care.^14-16^ It is also possible visits were missed as a result of dealing with illness at home or waiting for mandatory isolation periods before returning in person to a medical facility.^17,18^ Furthermore, non-English-speaking families may have found the additional telephone triage system particularly cumbersome.

Our finding that publicly insured pediatric patients were less likely to utilize telehealth may be influenced by the digital divide, wherein lower-income individuals are less likely to have broadband internet at home and more likely to rely on their phones for internet access.^19,20^ Clinical interactions on a small screen may be less satisfying or data limits with video calling may be constraints. We need better policies, both in our clinics and nationally, to ensure all pediatric patients to have equal access to telehealth.

Limitations of our study include the fact data were drawn from a single-site, urban pediatric academic practice, which may affect generalizability to other sites. Additionally, we did not examine failed telehealth visits (telephone visits) as there was not an easy method for distinguishing failed visits from no-shows, which could potentially mask digital access disparities. Finally, we were not able to assess our patients’ pediatric emergency room (ER) utilization between March and August 2020. We do know, however, that pediatric ER visits were significantly decreased throughout the United States during this time frame, so think it unlikely that our patients visited the ER instead of coming to our clinic.^21,22^ We expect our results would be generalizable to other urban academic pediatric primary care sites with a similar payor mix.

## Conclusion

Our study demonstrates that there were disparities in who accessed any type of care from our clinic during the first 5 months of the COVID-19 pandemic and that patients with public insurance were less likely to be seen with telehealth. These findings underscore the importance of clinical outreach to all patient populations, particularly those who have not been seen yet during the pandemic, to ensure they are obtaining the care they deserve. Telehealth is here to stay, but we must improve its use so it broadens, not restricts, patient access to care.

## Author Contributions

RBS: Contributed to conception or design; contributed to acquisition, analysis, or interpretation; drafted the manuscript; critically revised the manuscript; gave final approval; agrees to be accountable for all aspects of work ensuring integrity and accuracy.

MCL: contributed to conception or design; contributed to acquisition, analysis, or interpretation; drafted the manuscript; critically revised the manuscript; gave final approval; agrees to be accountable for all aspects of work ensuring integrity and accuracy.

AYO: Contributed to acquisition, analysis, or interpretation; critically revised the manuscript; gave final approval; agrees to be accountable for all aspects of work ensuring integrity and accuracy.

MJO: Contributed to conception or design; contributed to acquisition, analysis, or interpretation; critically revised the manuscript; gave final approval; agrees to be accountable for all aspects of work ensuring integrity and accuracy.

HB: Contributed to conception or design; contributed to acquisition, analysis, or interpretation; drafted the manuscript; critically revised the manuscript; gave final approval; agrees to be accountable for all aspects of work ensuring integrity and accuracy.
